# Long term disease free survival with multimodal therapy in small cell bladder cancer

**DOI:** 10.1186/s40001-016-0234-9

**Published:** 2016-10-13

**Authors:** Isabel Heidegger, Gennadi Tulchiner, Georg Schäfer, Wolfgang Horninger, Renate Pichler

**Affiliations:** 1 Department of Urology, Medical University Innsbruck, Anichstrasse 35, 6020 Innsbruck, Austria; 2 Division of General Pathology, Department of Pathology, Medical University Innsbruck, Innsbruck, Austria

**Keywords:** Small cell bladder cancer, Multimodal therapy, Neoadjuvant chemotherapy, Disease free survival

## Abstract

**Background:**

Small cell bladder cancer (SCBC) is an aggressive subtype accounting for less than 1 % of all bladder malignancies associated with rapid progression, early metastases formation and high mortality rates.

**Case presentation:**

We present an unusual long term disease free survival of a 60 year-old man who was diagnosed with SCBC two and a half years ago. He underwent four cycles of cisplatin/etoposide chemotherapy as well as a prophylactic whole-brain radiotherapy followed by a radical cystoprostatectomy and ileal neobladder with extended pelvic lymphadenectomy. Since 33 months the patient is now recurrence-free.

**Conclusion:**

In this case report, we were able to show that early multimodal therapy results in long term disease free survival, thus we highly recommend neoadjuvant chemotherapy as a part of multimodal management of a primary metastases-free, localized and surgically resectable SCBC.

## Background

Bladder cancer is one of the most common urological malignancies in men and women. While about 95 % of diagnosed bladder cancers are histologically urothelial cancers, small cell bladder cancer (SCBC) is an aggressive subtype accounting for less than 1 % of all bladder malignancies associated with rapid progression, early metastases formation and high mortality rates [1–3].

SCBC primarily affects caucasian males between 60 and 80 years, mostly with a history of heavy smoking. Clinical symptoms like hematuria, imaging and cystoscopy do not allow the prediction of this aggressive type of bladder cancer, thus only tissue diagnostics are able to diagnose SCBC. In the recent years, new histopathological markers including TMPSS-ERG fusions or a positive HPV status have been reported as risk factors for SCBC [4, 5].

As no general guidelines for the optimal treatment of SCBC are available in the urological field, single modality local therapy (15 %), surgical (21 %) or radiation-based (14 %) as well as multimodal therapies (50 %) including cisplatin-based chemotherapeutic regimes have been reported [5]. A recent investigation utilizing the National Cancer Data Base based on 960 SCBC patients revealed that median overall survival (OS) in patients who were metastasis free at primary diagnosis was 8.3 months [6].

We present an unusual long term disease free survival of a 60 year old man who was diagnosed with SCBC two and a half years ago. He underwent four cycles of cisplatin/etoposide chemotherapy as well as a prophylactic whole brain radiotherapy (WBRT) followed by a radical cystoprostatectomy and ileal neobladder with extended pelvic lymphadenectomy. Currently, the patient is recurrence-free since 33 months.

## Case report

A 60 years-old man was referred to our department due to painless gross hematuria. The patient had no risk factors including smoking, previous radiation therapy, occupational risk factors or hereditary factors. In addition, no other pre-existing conditions were known. The patient was painless and did not have any B-symptoms including weight loss or night sweats, furthermore, no neurological deficits were reported.

After exclusion of urinary infection as cause for hematuria, cystoscopy has been performed revealing a 6 cm solid tumor on the right bladder wall. Urinary cytology (voided urine and bladder washing) remained negative.

**Fig. 1:**
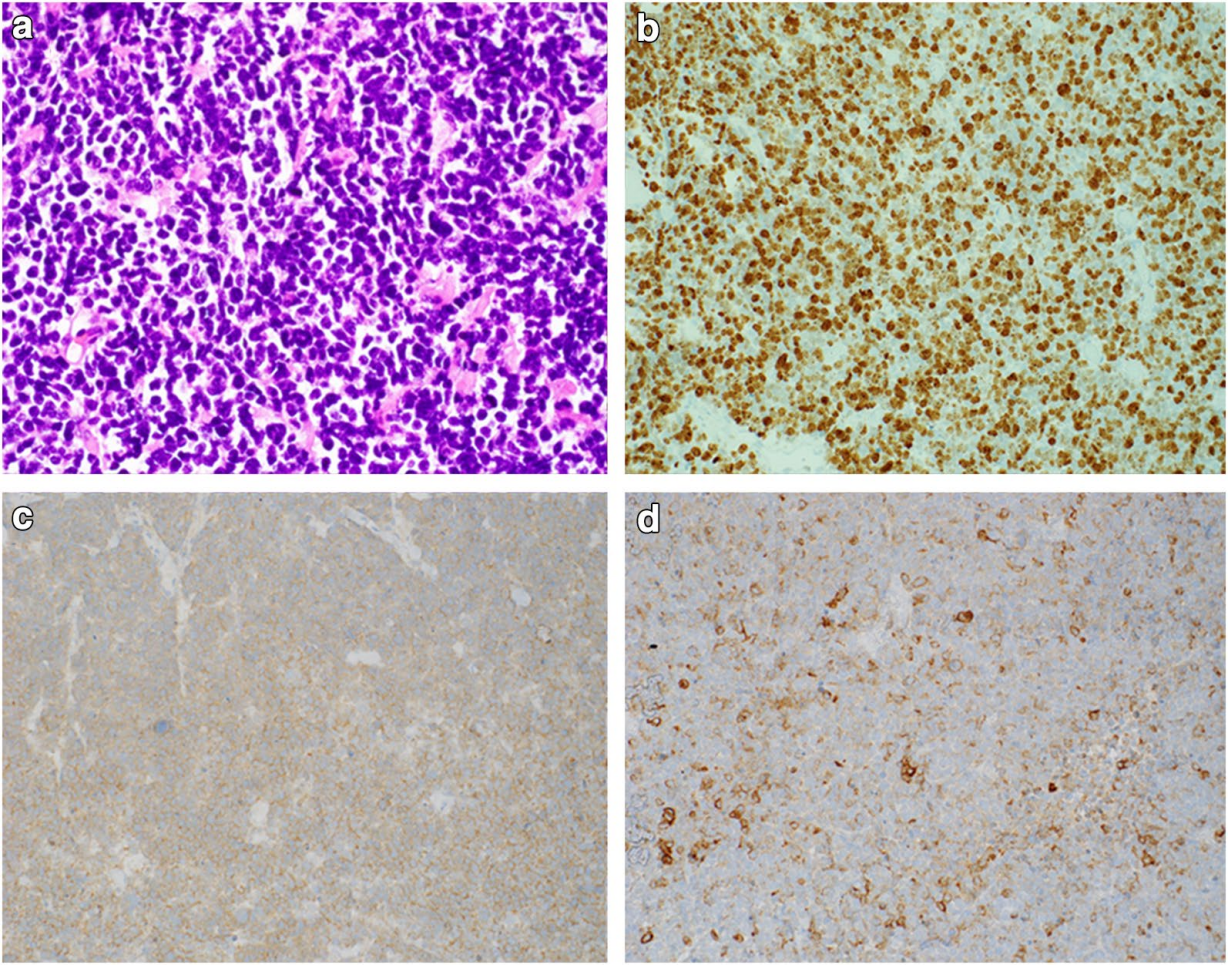
Hematoxylin and eosin staining of cancer tissue sections (**a**) and immunohistochemistry for KI-67 (**b**), synaptophysin (**c**) and AE1/AE3 (**d**)

Consequently, a transurethral resection of the tumor has been performed and the tumor was macroscopically totally removed. Primary histology of the tumor specimen showed a muscle invasive small cell neuroendocrine carcinoma pT2a GIII. Moreover, tumor cells were positive for synaptophysin and AE1/AE3, with a high proliferation rate (KI-67) of 95 % on immunohistochemical analysis (Fig. 1). In contrast, chromogranin A, CD56, CD3, CD20, TdT, S-100 and HMB45 confirmed negative staining.

18 FDG-positron emission tomography/computed tomography (PET/CT) performed at the time of primary diagnosis did not show any lymph node or visceral metastatic tumor spread. A subsequent cranial magnet resonance tomography (MRT) also confirmed no tumor infiltration into the brain.

As there are reports of increased survival rates upon neoadjuvant chemotherapy in patients suffering from SCBC [5], the patient underwent four cycles (day 1–3; 1 cycle = 21 days) of cisplatin (25 mg/m^2^)/etoposide (100 mg/m^2^) without any complications. In addition, we performed a prophylactic WBRT with a total dose of 26 Gray. Subsequently, the patient underwent radical cystoprostatectomy and ileal neobladder with bilateral extended pelvic lymphadenectomy (including 32 resected tumor-free lymph nodes). Final pathology confirmed complete response to neoadjuvant chemotherapy, with no vital small cell carcinoma tissue formations in both the lymph nodes and the cystoprostatectomy specimen (ypT0, N0, L0, V0, Pn0). An uneventful intra- and postoperative course was observed. The time from transurethral resection to chemotherapy start was 28 days. 34 days after chemotherapy was stopped, radical cystoprostatectomy has been performed.

Currently, the patient undergoes 6-monthly regular follow-up controls including urinary cytology (voided urine), measurement of residual urine, blood gas analysis and imaging studies (chest/abdominal CT scan every second visit or chest radiography in combination with abdominal ultrasound). We noticed no evidence for relapse, even 33 months after initial diagnosis of SCBC.

## Discussion

SCBC is a rare urological disease consequently associated with obvious limitations in literature and treatment experience leading to the fact that no clear urological-guideline based standard treatment is available for patients suffering from SCBC. To our knowledge, only the Canadian Association of Genitourinary Medical Oncologists recommended in 2013 treating the disease with neoadjuvant (Level 3, Grade C) or adjuvant chemotherapy (Level 4, Grade D) using cisplatin and etoposide (Level 3, Grade C), followed by cystectomy or radiation in a bladder-sparing therapy (Level 3, Grade C) [7].

Focusing on the literature, a multimodal treatment combining cisplatin-based neoadjuvant chemotherapy with radical surgery seems to be most efficacious, whereof chemotherapy has the key role in this therapy concept [8]. However, also bladder preservation multimodality approaches with a maximal complete transurethral resection, followed by radiation therapy and concurrent chemotherapy has demonstrated promising oncological outcomes [9]. Despite of this evidence, in the present case we decided to undergo radical cystoprostatectomy as the primary tumor has a size of 6 cm infiltrating almost the whole right bladder side-wall.

In addition, our patient underwent prophylactic whole-brain radiation as it has been reported that it may be considered and discussed with patients with limited and extensive disease who have had a good clinical response to treatment [7].

In general, the concept of neoadjuvant chemotherapy even if radiography lacks the evidence of metastatic disease, underlines the possibility to treat micrometastatic disease at an early stage and downstage the disease thereby facilitating radical surgery. In addition, as especially cisplatin correlates with numerous side effects, tolerability is higher before than after surgery.

Already 12 years ago Siefker-Radtke et al. [10] compared retrospectively the efficacy of neoadjuvant chemotherapy to surgery alone in a cohort of 46 patients. Thereby they found that the five years cancer specific survival was significantly higher in the chemotherapy group compared to those patients who underwent immediate surgery alone. A consequently performed prospective phase II clinical trial by the same group including 30 patients confirmed the survival benefit of neoadjuvant chemotherapy as part of multimodal treatment in SCBC [11].

In addition, there is evidence that neoadjuvant chemotherapy leads to pathological downstaging to ≤pT1N0 in 62 % of tumors compared to 9 % of patients treated with surgery alone. Our data are in line, or even better as these data, as pathology of the cystoprostatectomy specimen showed complete response, without any evidence for residual tumor after four neoadjuvant cycles of cisplatin/etoposide [12].

In general, SCBC primarily affects males aged between 60–80 years with a history of heavy smoking (reviewed in [13]). Interestingly, the present patient did not report a history of smoking, indicating that other risk factors induce genetic aberrations, mutations or epigenetic alterations. Thus, we feel the importance of further studies investigating this issue.

Reviewing the literature, median overall survival in patients who were metastasis-free at primary diagnosis was 8.3 months [5]. The patient presented in the manuscript has currently a disease-free OS of 33 months.

However, one has to consider that our case harbored an initial T2 GIII stage, with a complete response (pT0 N0) after neoadjuvant chemotherapy underlying the importance of a multimodal treatment concept. This might be of importance as Choong et al. [14] showed that higher stages are associated with poor outcome. On the other hand, our patient had a large tumor (6 cm) and—in line with urothelial cancers of the bladder tumor size >3 cm may be classified as a high risk tumor. Again, the fact that the patient had no smoking history possibly influences this extraordinary satisfactory outcome.

However, in summary we can assume that early multimodal treatment may help to diminish recurrence or progression rates in patients with SCBC.

## Conclusion

SCBC is a rare and very aggressive disease with a median overall survival rate of 8.3 months. Multimodal treatment has been shown to be superior to local therapy alone. Early multimodal therapy may result in long term disease free survival, thus we highly recommend neoadjuvant chemotherapy as a part of multimodal management of a primary metastases-free, localized and surgically resectable SCBC.

## Abbreviations

OS: overall survival
PET/CT: positron emission tomography/computed tomography
MRT: magnet resonance tomography
SCBC: small cell bladder cancer
WBRT: whole brain radiotherapy
